# Chalcogen‐Directed Dual‐Heterointerfaced Bi_2_Te_3_/BiS_x_ in N‐Doped Carbon Nanofibers for Sodium Storage

**DOI:** 10.1002/smll.73235

**Published:** 2026-03-26

**Authors:** Jinkai Wang, Min Wang, Hangcheng Yang, Zhengdong Wang, Yaoyao Wang, Gengrui Liu, Yiming Yong, Boshi Liu, Xinrui Zhou, Hongdu He, Hongkang Wang, Zongyou Yin

**Affiliations:** ^1^ School of Mechanical and Electrical Engineering Xi'an University of Architecture and Technology Xi'an China; ^2^ State Key Lab of Electrical Insulation and Power Equipment Center of Nanomaterials for Renewable Energy (CNRE) School of Electrical Engineering Xi'an Jiaotong University Xi'an China; ^3^ Research School of Chemistry Australian National University Canberra ACT Australia

**Keywords:** bismuth sulfide, bismuth telluride, crystalline/amorphous heterostructure, high‐energy‐density anodes, sodium‐ion batteries

## Abstract

Developing anode materials with both high volumetric capacity and robust cycling stability remains a critical challenge for sodium‐ion batteries (SIBs). Bismuth (Bi) offers promise due to its high theoretical volumetric capacity and suitable sodiation potential, yet its practical application is hindered by severe volume expansion during cycling. Herein, we report a rationally engineered dual heterointerfaced system of crystalline/amorphous Bi_2_Te_3_/BiS_x_ encapsulated in N‐doped porous carbon nanofibers (Bi_2_Te_3_/BiS_x_@NPCNFs), prepared via electrospinning coupled with in situ tellurization/sulfidation. The dual heterointerfaces—spanning both crystalline‐amorphous and chalcogen‐domain junctions of Bi_2_Te_3_/BiS_x_ synergistically enable fast charge transport, effective strain buffering, and stable structural integrity, while the conductive carbon matrix with Te vacancies further enhances electronic conductivity. As an anode for SIBs, the optimized Bi_2_Te_3_/BiS_x_@NPCNFs deliver exceptional performance, including a high reversible capacity (341.6 mAh g^−1^ at 0.1 A g^−1^), excellent rate capability (263.4 mAh g^−1^ at 2 A g^−1^), and long‐term cycling stability (288.7 mAh g^−1^ after 1000 cycles at 1 A g^−1^). Ex situ spectroscopy and microscopy elucidate the reversible phase evolution and sodium storage pathways, highlighting the critical role of dual heterointerfaces and defect engineering. This work establishes a general strategy for advancing high‐energy, long‐life sodium‐ion batteries by exploiting dual crystalline/amorphous and chalcogen heterointerfaces within nanostructured composites.

## Graphical abstract

Dual crystalline/amorphous heterointerfaces of Bi_2_Te_3_/BiS_x_ encapsulated in N‐doped porous carbon nanofibers are engineered as an advanced anode for sodium‐ion batteries. The synergistic heterointerfaces and Te vacancies enable fast charge transport, buffer volume expansion, and ensure structural integrity. The anode delivers exceptional rate capability and long‐term cycling stability, achieving a high energy density in full cells.

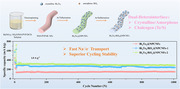

## 1 Introduction

The global transition toward carbon neutrality has intensified the demand for sustainable and cost‐effective energy storage solutions [1]. Sodium‐ion batteries (SIBs) have emerged as compelling alternatives to lithium‐ion systems for large‐scale energy storage, owing to the natural abundance of sodium and their potential for lower production costs [2, 3]. However, the development of high‐performance SIBs is hindered by the substantial volume expansion and structural degradation of anode materials caused by the larger ionic radius of Na^+^ (1.02 Å) compared to Li^+^ (0.76 Å) during repeated cycling [4, 5]. Addressing this challenge requires advanced anode materials that simultaneously deliver high specific capacity, long‐term cycling stability, and superior rate capability.

Among the diverse SIB anode materials—ranging from carbon‐based (intercalation‐type) and alloy‐type materials to conversion‐type metal oxides/sulfides and organic compounds [6, 7, 8] —bismuth (Bi)‐based materials stand out due to their high theoretical capacity (385 mAh g^−1^ and 1075 mAh cm^−3^), suitable operating potential (less than 1.0 V vs. Na^+^/Na), and excellent electrical conductivity [9, 10]. The alloying mechanism of Bi with Na to form Na_3_Bi offers additional advantages, including a smooth voltage profile and high volumetric capacity. Nevertheless, severe volume changes during sodiation/desodiation lead to electrode pulverization and rapid capacity decay, limiting practical implementation [11].

To mitigate these issues, two key strategies have been pursued: (i) constructing carbon‐based composites to buffer volume expansion and enhance electronic conductivity [12], and (ii) designing nanostructured materials to shorten ion diffusion paths and alleviate mechanical stress [13]. Bismuth telluride (Bi_2_Te_3_), a layered semiconductor with unique van der Waals gaps, has recently gained attention as a promising SIB anode, as its interlayer spacing can accommodate Na^+^ while maintaining structural integrity [14]. Notably, introducing polyvinylpyrrolidone (PVP) during synthesis creates hierarchical porous architectures and Te vacancies, significantly enhancing Na^+^ storage [15]. The polar groups (N–C = O) in PVP interact with inorganic crystals via spatial confinement and electrostatic effects, enabling precise morphology control and defect engineering [16, 17].

Previous studies have illuminated both the promise and limitations of Bi_2_Te_3_‐based anodes. Gillarda et al. reported rapid capacity decay (43% after 10 cycles) in solid‐state synthesized Bi_2_Te_3_ [18]. Sun et al. demonstrated improved performance through compositing with graphene, achieving 416 mAh g^−1^ at 0.1 A g^−1^, albeit with a complex synthesis process [19]. Cui et al. used a hydrothermal method to fabricate Bi_2_Te_3_ van der Waals layered nanoplate coated with a thin uniform polypyridine layer [20]. These efforts underscore the need for innovative material design to optimize the electrochemical performance of Bi_2_Te_3_‐based anodes.

Electrospinning has emerged as a powerful technique for fabricating advanced electrode architectures, offering high surface‐to‐volume ratios, tunable porosity, and exceptional material versatility [21]. The resulting nanofiber morphology can significantly enhance electrode kinetics by providing shortened ion diffusion pathways and abundant electrochemically active sites [22, 23]. In this work, we developed a novel synthetic strategy combining electrospinning with precisely controlled thermal processing to fabricate Te vacancy‐rich Bi_2_Te_3_ encapsulated within N‐doped porous carbon nanofibers (Bi_2_Te_3_@NPCNFs). Furthermore, through an additional sulfurization process, we successfully constructed crystalline/amorphous Bi_2_Te_3_/BiS_x_ heterostructures within the carbon matrix (Bi_2_Te_3_/BiS_x_@NPCNFs), which exhibit remarkable synergistic enhancement in both specific capacity and cycling stability. Systematic characterization and electrochemical analysis reveal the underlying mechanisms for the improved performance, offering new insights into defect and interface engineering for advanced bismuth‐based SIB anodes.

## 2 Results and Discussion

**FIGURE 1:**
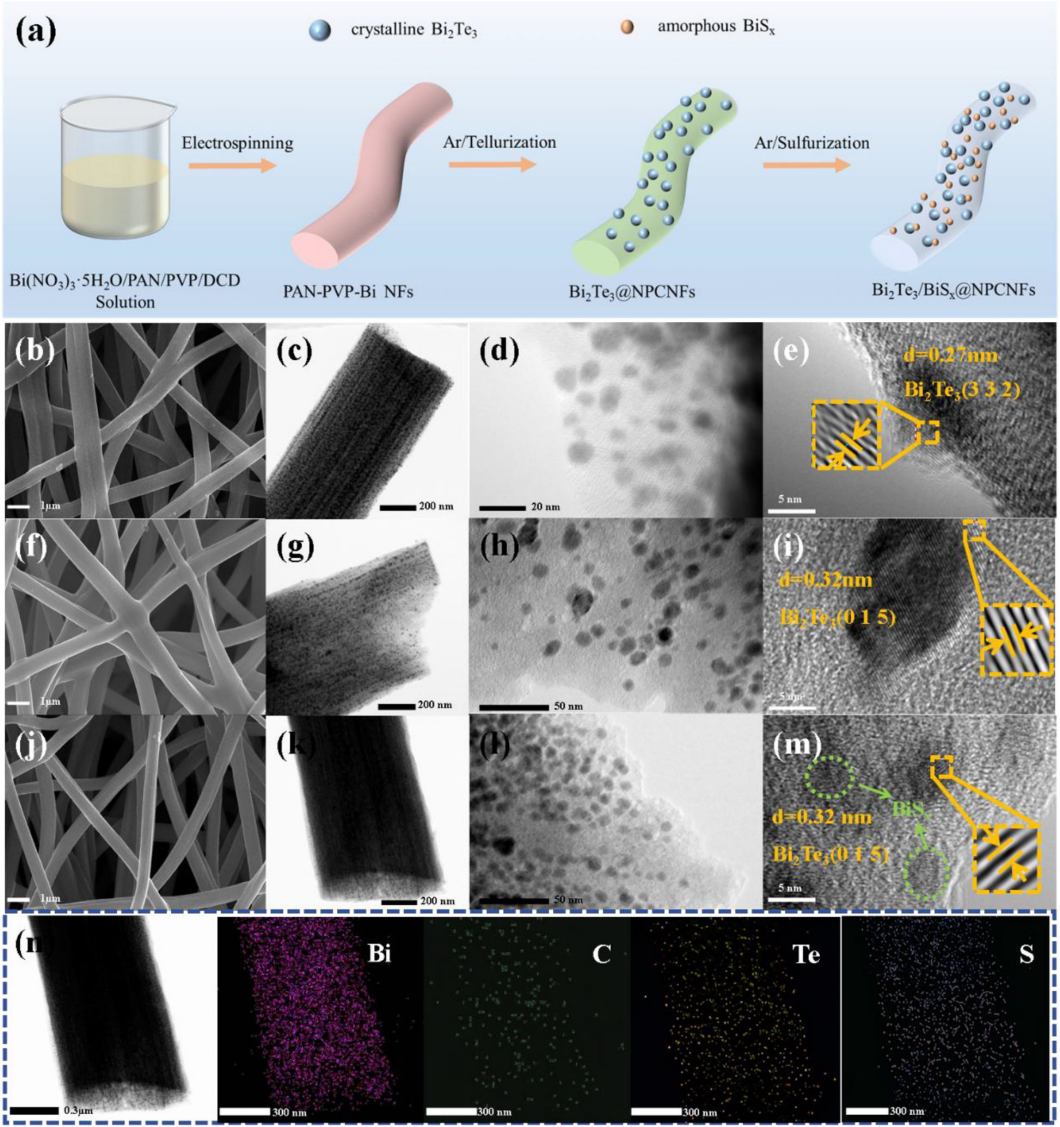
(a) Schematic illustration for the synthesis of Bi_2_Te_3_/BiS_x_@NPCNFs. (b), (f), and (j) SEM images, (c), (d), (g), (h), and (k), (l) TEM images, and (e), (i), and (m) HRTEM images of Bi_2_Te_3_@NPCNFs, Bi_2_Te_3_/BiS_x_@NPCNFs‐1, and Bi_2_Te_3_/BiS_x_@NPCNFs‐2. (n) EDX elemental mappings of Bi_2_Te_3_/BiS_x_@NPCNFs‐2.

Figure 1a illustrates the synthetic route for constructing N‐doped porous carbon nanofiber‐supported Bi_2_Te_3_/BiS_x_ heterostructured composites (Bi_2_Te_3_/BiS_x_@NPCNFs) through an electrospinning, PVP‐etching, and in situ telluride/sulphide processes. The PAN‐PVP‐Bi precursor nanofibers were first prepared via electrospinning, followed by carbonization and in situ tellurization at 600°C under an Ar atmosphere to yield uniformly distributed Bi_2_Te_3_ nanoparticles embedded in N‐doped porous carbon nanofibers (Bi_2_Te_3_@NPCNFs). Notably, the synergistic effect between the porous carbon matrix and precisely controlled reduction conditions facilitates the formation of abundant Te vacancies. Finally, the crystalline Bi_2_Te_3_/amorphous BiS_x_ heterostructures with tunable sulfide degree in Bi_2_Te_3_/BiS_x_@NPCNFs were successfully constructed by regulating the sulfur dosage during sulfurization treatment at 600°C.

The morphological evolution of the materials was systematically investigated using scanning electron microscopy (SEM), transmission electron microscopy (TEM), and high‐resolution TEM (HRTEM). As illustrated in Figure S1, the Bi_2_Te_3_@NCNFs samples without added PVP showed the morphological characteristics of straight fibers, rough surface, and the presence of large particles. In contrast, the PVP‐modified Bi_2_Te_3_@NPCNFs samples (Figure 1b,c) exhibited a 3D fibrous network structure with a diameter of 200–300 nm, a relatively smooth surface, and uniformly distributed nanoparticles, which fully proved the key role of PVP in the morphology modulation. Remarkably, after sulfidation treatment, the obtained crystalline/amorphous Bi_2_Te_3_/BiS_x_@NPCNFs heterostructures (Figure 1f,j) still maintain excellent structural integrity, and the comparative analyses (Figure 1g,k) further confirm their outstanding stability. These results clearly demonstrate that PVP‐derived hierarchical porosity not only improves the surface smoothness of the material but also significantly increases the specific surface area [24, 25].

High‐resolution transmission electron microscopy (HRTEM) analysis provides conclusive evidence for the formation of heterostructures. The pristine Bi_2_Te_3_@NPCNFs sample exhibits characteristic (332) lattice fringes (d = 0.27 nm, PDF#85‐0439, Figure 1e). For Bi_2_Te_3_/BiS_x_@NPCNFs, HRTEM reveals the coexistence of crystalline (d = 0.32 nm, (015) plane, PDF#08‐0027) and amorphous domains (Figure 1i,m) [26], directly confirming the successful formation of the crystalline/amorphous heterostructures. The elemental distribution maps (Figure 1n) demonstrate the homogeneous distribution of Bi, C, Te, and S elements throughout the composite, revealing the strong interfacial coupling between Bi_2_Te_3_ and BiS_x_ phases within the heterostructure.

**FIGURE 2:**
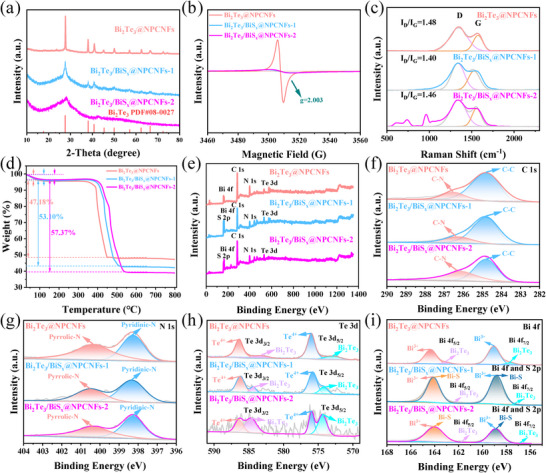
(a) XRD patterns, (b) EPR curves, (c) Raman spectra, and (d) TGA curves of Bi_2_Te_3_@NPCNFs, Bi_2_Te_3_/BiS_x_@NPCNFs‐1, and Bi_2_Te_3_/BiS_x_@NPCNFs‐2. XPS spectra of (e) Survey spectra, (f) C 1s, (g) N 1s, (h) Te 3d, and (i) Bi 4f and S 2p of Bi_2_Te_3_@NPCNFs, Bi_2_Te_3_/BiS_x_@NPCNFs‐1, and Bi_2_Te_3_/BiS_x_@NPCNFs‐2.

X‐ray diffraction (XRD) analysis was systematically performed to investigate the structural evolution of the synthesized materials. As revealed in Figure S2a, the diffraction pattern of Bi_2_Te_3_@NCNFs can be precisely indexed to the Bi_4_Te_3_ phase (PDF#33‐0216) coexisting with metallic Bi phase (PDF#85‐1330). In contrast, the Bi_2_Te_3_@NPCNFs composite (Figure 2a) displays well‐defined diffraction peaks at 17.7°, 27.6°, 38.1°, 40.8°, and 45.4°, corresponding to the (006), (015), (1010), (110), and (0015) crystal planes of rhombohedral Bi_2_Te_3_ phase (PDF#08‐0027), respectively. The absence of impurity peaks confirms the high purity of the synthesized products. Notably, the diffraction peaks of Bi_2_Te_3_@NPCNFs show a positive shift compared to Bi_2_Te_3_@NCNFs, which can be attributed to the generation of Te vacancies during the high‐temperature tellurization process. Of particular significance is the exceptionally large interplanar spacing associated with the dominant (015) crystal plane in Bi_2_Te_3_, which provides favorable channels for Na^+^ insertion/extraction and contributes to the excellent reversible cycling performance. Furthermore, XRD analysis of the Bi_2_Te_3_/BiS_x_@NPCNFs composite demonstrates similar diffraction patterns to Bi_2_Te_3_@NPCNFs, while the amorphous nature of BiS_x_ is confirmed by the broad diffraction hump shown in Figure S6a. These results provide convincing evidence for the successful construction of crystalline Bi_2_Te_3_/amorphous BiS_x_ heterostructures.

Electron paramagnetic resonance (EPR) spectroscopy was employed to probe the defect states in the synthesized materials, as presented in Figure 2b. A prominent EPR signal centered at g = 2.003 was clearly observed, which is characteristic of abundant Te vacancies in the Bi_2_Te_3_@NPCNFs composite [27, 28]. The hierarchical porous architecture of Bi_2_Te_3_@NPCNFs facilitates efficient exposure to the reducing atmosphere during high‐temperature annealing, thereby promoting the generation of Te vacancies through controlled defect engineering. Notably, the EPR signal intensity associated with Te vacancies exhibited significant attenuation in the Bi_2_Te_3_/BiS_x_@NPCNFs heterostructure. This phenomenon can be attributed to two possible mechanisms: (i) sulfur atoms may occupy the original Te lattice sites during the sulfurization process, thereby directly reducing the Te vacancy concentration; (ii) according to chemical equilibrium principles, the introduction of sulfur may alter the thermodynamic equilibrium during crystal growth/annealing, increasing the formation energy of Te vacancies and consequently suppressing their generation [29]. These results provide direct evidence for the effective modulation of defect engineering through the sulfurization treatment.

Raman spectroscopic analysis provides important insights into the structural characteristics of the prepared materials. As depicted in Figure 2c, the Raman spectra exhibit two characteristic peaks at 1343.8 cm^−1^ (D band) and 1568.0 cm^−1^ (G band), corresponding to disordered carbon and graphitic carbon, respectively [30]. The intensity ratio of these bands (I_D_/I_G_) serves as a quantitative indicator of the graphitization degree, with higher values indicating more structural defects or lower graphitization. Notably, Bi_2_Te_3_@NPCNFs (I_D_/I_G_ = 1.48), Bi_2_Te_3_@NCNFs (I_D_/I_G_ = 1.56, Figure S2b), Bi_2_Te_3_/BiS_x_@NPCNFs‐1 (I_D_/I_G_ = 1.40), and Bi_2_Te_3_/BiS_x_@NPCNFs‐2 (I_D_/I_G_ = 1.46) composites have exhibit comparable I_D_/I_G_ ratios, demonstrating that all Bi_2_Te_3_‐based composites maintain similar structural quality and graphitization degree despite undergoing different chemical modifications.

Thermogravimetric analysis (TGA) was performed to elucidate the compositional evolution and thermal stability of the synthesized materials (Figure 2d; Figure S2c). The carbon content in Bi_2_Te_3_@NPCNFs (47.2 wt.%) was notably higher than that in Bi_2_Te_3_@NCNFs (45.4 wt.%), demonstrating that PVP addition effectively enhances the carbonization efficiency. Notably, the sulfurized samples exhibited substantially enhanced carbon contents along with significantly improved thermal stability. Specifically, the Bi_2_Te_3_/BiS_x_@NPCNFs‐1 and Bi_2_Te_3_/BiS_x_@ NPCNFs‐2 composites demonstrated increased carbon contents of 53.1 and 57.4 wt.%, respectively. Concurrently, these sulfur‐incorporated samples displayed a notable ∼50°C elevation in thermal decomposition temperature, underscoring the dual benefits of sulfurization. This synergistic improvement can be attributed to two predominant mechanisms: (i) sulfur‐induced crosslinking within the carbon matrix that reinforces structural integrity, and (ii) formation of thermally robust bismuth sulfotelluride phases that effectively mitigate metal component loss during thermal treatment.

X‐ray photoelectron spectroscopy (XPS) was employed to investigate the surface chemical composition and electronic states of the prepared materials. The survey spectrum of Bi_2_Te_3_@NPCNFs (Figure 2e) clearly reveals the presence of C, N, Bi, and Te elements, while additional S signals are detected in the Bi_2_Te_3_/BiS_x_@NPCNFs composites, verifying the successful sulfur incorporation. High‐resolution C 1s spectra (Figure 2f) exhibit two characteristic peaks at 284.8 and 286.5 eV, corresponding to C‐N and C‐C bonds, respectively. The N 1s spectra (Figure 2g) can be deconvoluted into two components at 398.3 eV (pyridinic N) and 400.4 eV (pyrrolic N), confirming successful nitrogen doping in the carbon matrix. The Te 3d spectrum of Bi_2_Te_3_@NPCNFs (Figure 2h) exhibits two peaks at 573.0 eV (Te 3d_5/2_) and 583.3 eV (Te 3d_3/2_), while the two peaks at 576.0 eV and 586.4 eV correspond to the oxide layer on the material's surface [31]. Remarkably, the Te 3d peaks in Bi_2_Te_3_/BiS_x_@ NPCNFs‐2 show a positive shift to higher binding energies (574.4 eV for Te 3d_5/2_ and 584.7 eV for Te 3d_3/2_) compared to Bi_2_Te_3_@NPCNFs, indicating altered electronic environments due to heterostructure formation. Similarly, the Bi 4f spectrum of Bi_2_Te_3_/BiS_x_@NPCNFs‐2 (Figure 2i) displays peaks at 156.9 eV (Bi 4f_7/2_) and 161.8 eV (Bi 4f_5/2_), which are slightly negatively shifted from those of Bi_2_Te_3_@NPCNFs (157.7 eV and 163.0 eV). Besides, the other two peaks at 158.9 and 164.0 eV are attributed to Bi 4f_7/2_ and Bi 4f_5/2_ of oxide on the surface of the Bi_2_Te_3_/BiS_x_@NPCNFs‐2 sample [32, 33]. The S 2p spectrum (Figure 2i) further confirms the presence of Bi‐S bonds through characteristic peaks overlapping with Bi_2_O_3_ [34, 35]. These systematic shifts in binding energies provide strong evidence for the successful construction of Bi_2_Te_3_/BiS_x_ heterostructures, which are expected to enhance reaction kinetics during rapid Na^+^ storage processes [36].

The electrochemical reaction mechanisms of both Bi_2_Te_3_@NPCNFs and Bi_2_Te_3_/BiS_x_@NPCNFs electrodes were systematically investigated through cyclic voltammetry analysis at a scan rate of 0.2 mV/s. For Bi_2_Te_3_@NPCNFs (Figure S3b), the initial discharge process exhibits a characteristic reduction peak at 1.39 V, corresponding to Na^+^ intercalation into the Bi_2_Te_3_ lattice to form Na_x_Bi_2_Te_3_ alongside the formation of the solid electrolyte interphase (SEI) [37]. Subsequently, two consecutive peaks (1.05 V and 0.32 V) appear, attributed respectively to the conversion reaction (Na_x_Bi_2_Te_3_→Bi+Na_2_Te) and the subsequent two‐step alloying reaction (Bi→NaBi→Na_3_Bi). During charging, the electrode demonstrates excellent reversibility through well‐defined oxidation peaks at 0.71/0.81 V (dealloying: Na_3_Bi→NaBi→Bi) and 1.70 V (reconversion: Bi+Na_2_Te→Bi_2_Te_3_). The slight positive shift of oxidation peaks in subsequent cycles suggests progressive electrode activation through cycling‐induced particle refinement and improved reaction kinetics. Notably, the CV curves of the Bi_2_Te_3_@NPCNFs electrode overlap well with minimal peak potential drift after 5 cycles, demonstrating exceptional electrochemical reversibility and structural stability.

**FIGURE 3:**
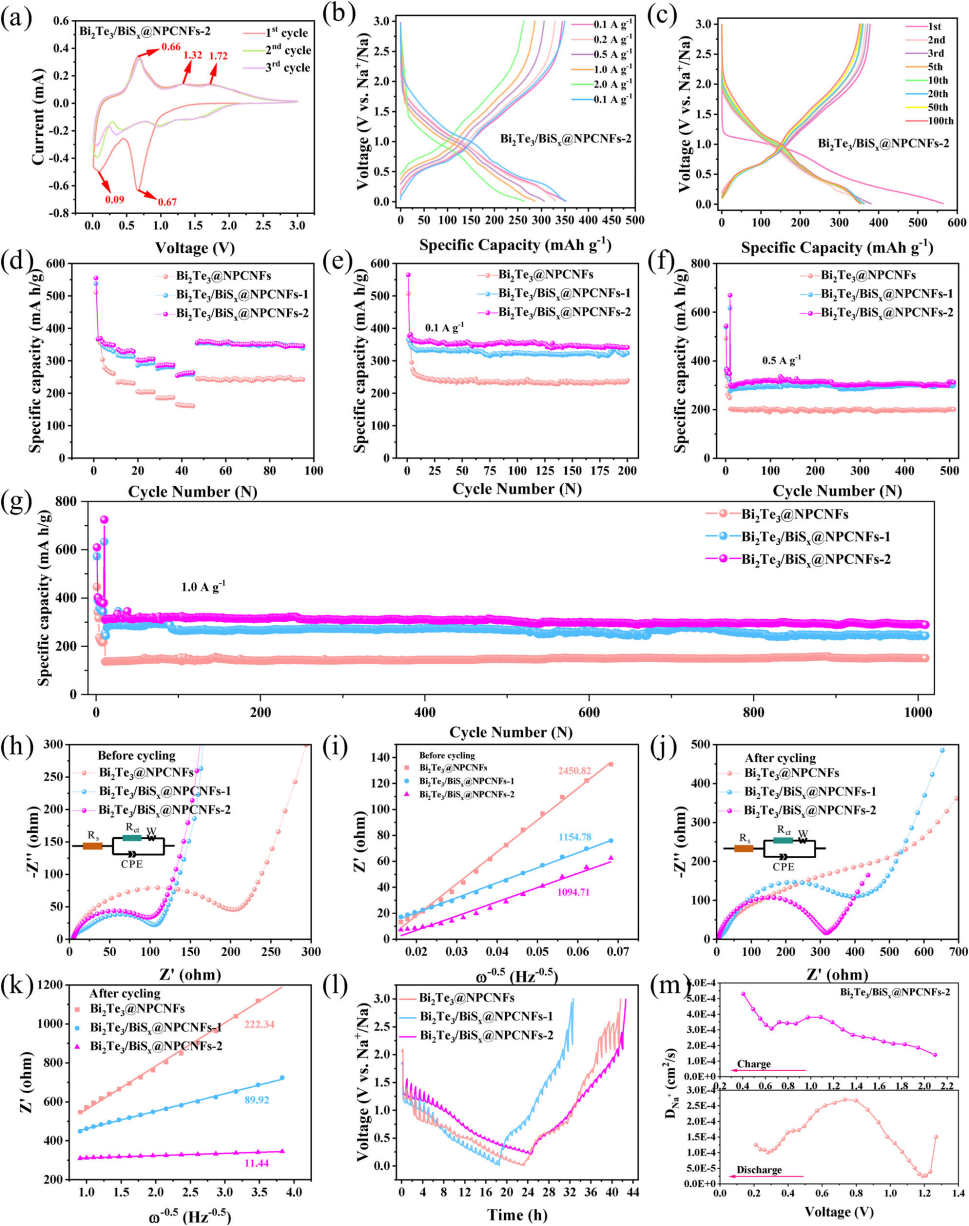
(a) CV curves at a scan rate of 0.2 mV s^−1^ for the first, second, and third cycles, (b) Charge/discharge curves at different current densities, and (c) The charge–discharge profiles at a current density of 0.1 A g^−1^ of Bi_2_Te_3_/BiS_x_@ NPCNFs‐2 electrode. (d) Rate performances at various currents ranging from 0.1 to 2.0 A g^−1^. (e), (f) The cycling performance at 0.1 A g^−1^ and 0.5 A g^−1^, (g) The long‐term cycling performance at the current density of 1.0 A g^−1^ of Bi_2_Te_3_@NPCNFs, Bi_2_Te_3_/BiS_x_@NPCNFs‐1, and Bi_2_Te_3_/BiS_x_@NPCNFs‐2 electrodes. (h) and (j) Nyquist plots and (i) and (k) corresponding results of EIS calculations before cycling and after 10 cycles at 0.1 A g^−1^ of Bi_2_Te_3_@NPCNFs, Bi_2_Te_3_/BiS_x_@NPCNFs‐1 and Bi_2_Te_3_/BiS_x_@NPCNFs‐2 electrodes. (l) GITT profiles of Bi_2_Te_3_@NPCNFs, Bi_2_Te_3_/BiS_x_@NPCNFs‐1 and Bi_2_Te_3_/ BiS_x_@NPCNFs‐2 electrodes. (m) The calculated diffusion coefficient of Bi_2_Te_3_/BiS_x_@NPCNFs‐2 electrode during discharge and charge process.

Similarly, the Bi_2_Te_3_/BiS_x_@NPCNFs electrode (Figure 3a; Figure S3c) displays distinct electrochemical behavior during initial cycling. The cathodic scan shows a composite peak at 0.67 V, representing the simultaneous Na^+^ insertion into BiS_x_ to form Na_y_BiS_x_ [38], subsequent conversion reaction (Na_y_BiS_x_→Bi+Na_y_S), and SEI formation [39], followed by a two‐step alloying process at 0.09 V (2Bi+Na^+^+e^−^→NaBi_2_; NaBi_2_+ 5Na^+^+5e^−^→2Na_3_Bi) [2]. The anodic scan reveals a dominant dealloying peak at 0.66 V (Na_3_Bi→Bi) [4, 6] accompanied by two reconversion peaks at 1.32 V and 1.72 V $\left(Bi+Na_{y}S\to Na_{y}BiS_{x}+Na^{+}+e^{-}\right)$. The remarkable overlap of CV curves in subsequent cycles with minimal peak shifts confirms the outstanding electrochemical reversibility and structural stability of these heterostructured electrodes, attributable to the synergistic stabilization effects of the crystalline/amorphous interfaces and conductive carbon matrix.

The galvanostatic charge–discharge (GCD) profiles of Bi_2_Te_3_@NPCNFs, Bi_2_Te_3_/BiS_x_@NPCNFs‐1, and Bi_2_Te_3_/BiS_x_@NPCNFs‐2 electrodes at various current densities (0.1‐2 A g^−1^) are presented in Figure 3b and Figure S3e,f, which demonstrates well‐defined voltage plateaus corresponding to reversible Na^+^ storage reactions. Notably, the heterostructured Bi_2_Te_3_/BiS_x_@NPCNFs electrode exhibits significantly reduced polarization even at elevated current densities, indicative of enhanced reaction kinetics. Besides, the GCD curves for the first, second, third, fifth, 10th, 20th, 50th, and 100th cycles at a current density of 0.1 A g^−1^ (Figure 3c; Figure S3h,i) reveal exceptional stability, with all electrodes maintaining consistent voltage profiles beyond the third cycle and minimal changes in plateau characteristics [40, 41]. The Bi_2_Te_3_/BiS_x_@NPCNFs‐2 electrode delivers outstanding electrochemical performance, achieving both the highest initial coulombic efficiency (97.1%) and capacity retention (94.2% after 100 cycles). The measured reversible capacities of 260.6, 328.6, and 348.2 mA h g^−1^ for Bi_2_Te_3_@NPCNFs, Bi_2_Te_3_/BiS_x_@NPCNFs‐1, and Bi_2_Te_3_/BiS_x_@NPCNFs‐2 correspond to 67.7%, 88.4%, and 90.4% of their theoretical capacity (385 mA h g^−1^), respectively, demonstrating efficient active material utilization.

Figure 3d demonstrates the outstanding rate capability of Bi_2_Te_3_/BiS_x_@NPCNFs‐2, delivering reversible capacities of 351, 327.5, 301.1, 286.2, and 261.2 mAh g^−1^ at current densities of 0.1, 0.2, 0.5, 1.0, and 2.0 A g^−1^, respectively. Remarkably, when returning to 0.1 A g^−1^, the Bi_2_Te_3_/BiS_x_@NPCNFs‐2 electrode still maintains 96.7% of its initial capacity, demonstrating exceptional electrochemical reversibility and minimal polarization. This rate performance significantly surpasses that of Bi_2_Te_3_@NPCNFs, which is attributed to the optimized crystalline/amorphous heterointerface, facilitating the fast charge transfer kinetics.

In addition, the cycling performances of Bi_2_Te_3_@NPCNFs, Bi_2_Te_3_/BiS_x_@ NPCNFs‐1, and Bi_2_Te_3_/BiS_x_@NPCNFs‐2 electrodes at current densities of 0.1 and 0.5 A g^−1^ are shown in Figure 3e,f. At 0.1 A g^−1^ (Figure 3e), the Bi_2_Te_3_/BiS_x_@NPCNFs‐2 electrode displays the superior cycling performance, maintaining a high specific capacity of 341.6 mAh g^−1^ after 100 cycles. The performance advantage becomes even more pronounced at higher current densities, with Bi_2_Te_3_/BiS_x_@NPCNFs‐2 delivering 312.1 mAh g^−1^ after 500 cycles at 0.5 A g^−1^, which exceeds that of the Bi_2_Te_3_@NPCNFs electrode (201.0 mAh g^−1^) and the Bi_2_Te_3_/BiS_x_@NPCNFs‐1 electrode (300.7 mAh g^−1^) (Figure 3f). In addition, Figure 3g displays the long‐term cycling performance of Bi_2_Te_3_@NPCNFs and Bi_2_Te_3_/BiS_x_@NPCNFs electrodes at a high current density of 1.0 A g^−1^. The Bi_2_Te_3_/BiS_x_@NPCNFs‐2 electrode achieves a high initial capacity of 402.7 mAh g^−1^ with a capacity decay rate of 0.028% per cycle after 1000 cycles, which exhibits high specific capacity, superior rate capability, and excellent long‐term cyclic stability than those of Bi_2_Te_3_@NCNFs (97.1 mAh g^−1^ in Figure S4d), Bi_2_Te_3_@NPCNFs (150.4 mAh g^−1^), and Bi_2_Te_3_/BiS_x_@NPCNFs‐1 (243.6 mAh g^−1^) (Figure 3g) electrodes at 1 A g^−1^. Meanwhile, as shown in Table S1, the reversible capacity and cycling performance of the Bi_2_Te_3_/BiS_x_@NPCNFs‐2 electrode are superior to those of previously reported Bi‐based anodes for SIBs, which is due to that the nitrogen‐doped carbon nanofibers and the Bi_2_Te_3_/BiS_x_ heterogeneous interfaces in the Bi_2_Te_3_/BiS_x_@NCNFs electrode play a key role. The excellent electrochemical properties can be attributed to the following synergistic effects: (1) the hierarchical porous architecture enables efficient electrolyte penetration and Na^+^ diffusion, (2) the nitrogen‐doped carbon network provides continuous electron transport pathways, and (3) the crystalline/amorphous heterointerface promotes interfacial charge storage and structural stability during prolonged cycling.

The electrochemical impedance spectroscopy (EIS) results provide critical insights into the interfacial charge transfer characteristics of the electrode materials. The Nyquist plots (Figure 3h) reveal distinct charge transfer resistance (R_ct_) values of 202 Ω, 105 Ω, and 98 Ω for the pristine Bi_2_Te_3_@NPCNFs, Bi_2_Te_3_/BiS_x_@NPCNFs‐1, and Bi_2_Te_3_/BiS_x_@NPCNFs‐2 electrodes, respectively, demonstrating the superior charge transfer kinetics of the heterostructured materials in their initial state. After 10 cycles at 0.1 A g^−1^ (Figure 3j), the R_ct_ values increase to 547 Ω, 398 Ω, and 317 Ω for the respective electrodes, reflecting a general deceleration of interfacial charge transfer processes during electrochemical cycling. Notably, despite this increase, the Bi_2_Te_3_/BiS_x_@NPCNFs‐2 electrode maintains the most favorable charge transfer characteristics compared to Bi_2_Te_3_@NPCNFs and Bi_2_Te_3_/BiS_x_@NPCNFs‐1, confirming its excellent charge transport capability of the optimized heterostructure [42].

Besides, the values of the Warburg diffusion coefficients for the Bi_2_Te_3_@NPCNFs and Bi_2_Te_3_/BiS_x_@NPCNFs electrodes before and after cycling are shown in Figure 3i,k. Based on the relationship between the frequency (ω^−1/2^) and the real part of the impedance (Z′) in the low‐frequency region, it can be seen that the Bi_2_Te_3_/BiS_x_@NPCNFs‐2 electrode demonstrates a steeper Warburg slope compared to Bi_2_Te_3_/BiS_x_@NPCNFs and Bi_2_Te_3_/BiS_x_@NPCNFs‐1 both before and after cycling, corresponding to a lower diffusion resistance and a reduction in charge transfer resistance [43, 44]. Comprehensive EIS tests and diffusion coefficient analysis confirm that the crystalline/amorphous heterostructure design enhances the interfacial kinetics and structural stability, further explaining the excellent electrochemical performance observed in the Bi_2_Te_3_/BiS_x_@NPCNFs system.

The galvanostatic intermittent titration technique (GITT) profiles and corresponding sodium ion diffusion coefficients (D_Na_^+^) of the Bi_2_Te_3_@NPCNFs and Bi_2_Te_3_/BiS_x_@NPCNFs electrodes during charge/discharge processes are presented in Figure 3l, m. As anticipated, the Bi_2_Te_3_/BiS_x_@NPCNFs‐2 electrode demonstrate superior sodium‐ion diffusion characteristics, with D_Na+_ values ranging from 2.02 × 10^−12^ to 5.50 × 10^−12^ cm^2^ s^−1^ during charging and 3.69 × 10^−13^ to 3.88 × 10^−12^ cm^2^ s^−1^ during discharging, significantly outperforming all other comparative samples. These extraordinary diffusion kinetics clearly indicate that the rationally designed crystalline/amorphous heterostructures in the Bi_2_Te_3_/BiS_x_@NPCNFs electrodes efficiently contribute to the enhancement of the ionic diffusion pathway. This structural feature greatly improves the reaction kinetics through rapid ion transport and excellent electrochemical reversibility.

**FIGURE 4:**
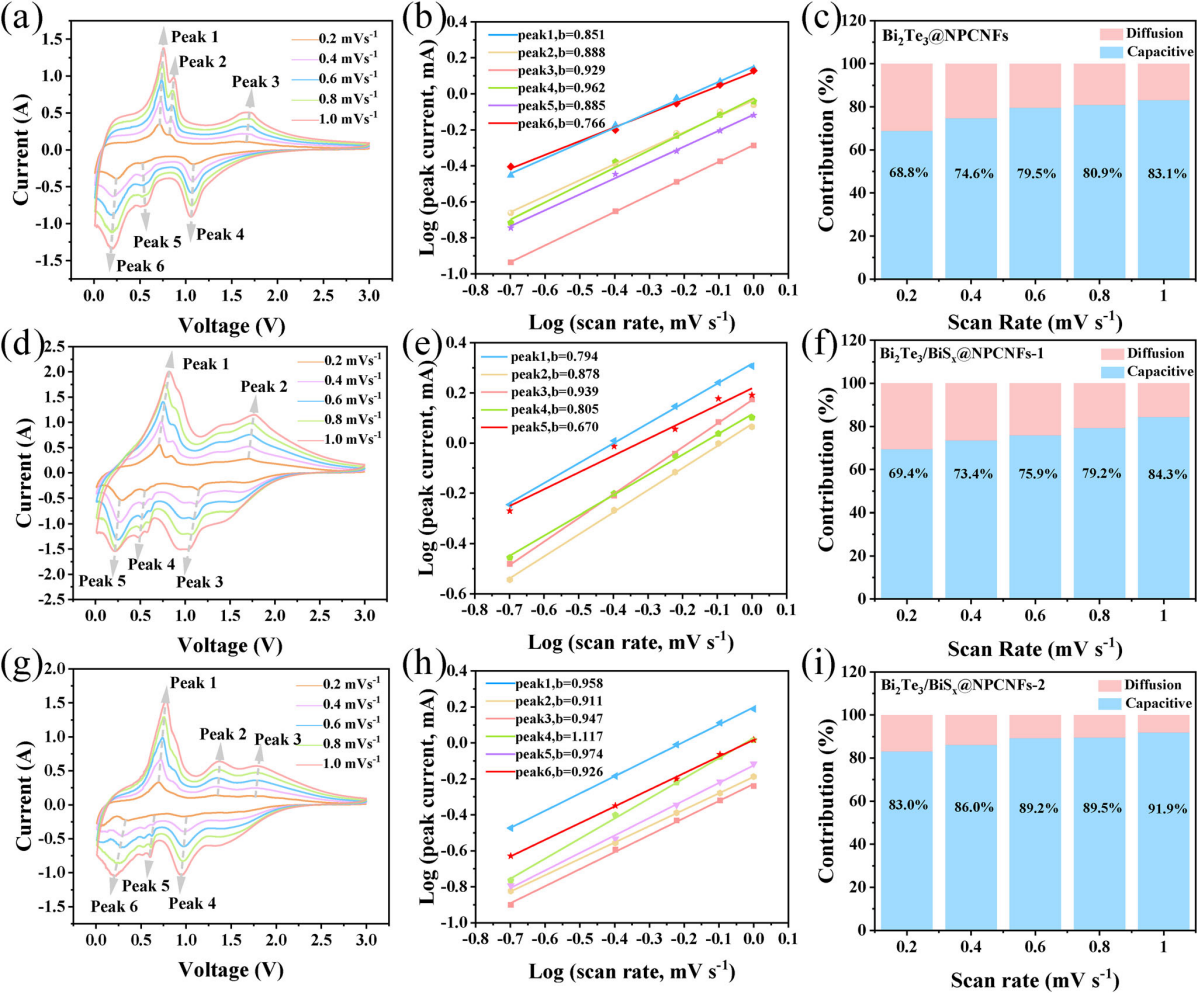
CV curves at different scan rates, calculated b values from CV curves, and capacitive contribution at different scan rates of (a–c) Bi_2_Te_3_@NPCNFs, (d–f) Bi_2_Te_3_/BiS_x_@NPCNFs‐1, (g–i) Bi_2_Te_3_/BiS_x_@NPCNFs‐2 electrodes.

The sodium storage behaviors of the as‐prepared Bi_2_Te_3_@NPCNFs and Bi_2_Te_3_/BiS_x_@NPCNFs electrodes were systematically investigated through cyclic voltammetry (CV) at varying scan rates (0.2–1.0 mV/s). As depicted in Figure 4a,d, and g and Figure S5a, all the CV curves exhibit similar redox characteristics, and the peak currents increase as the scan rate increases, confirming pseudocapacitance behavior [45]. Notably, the Bi_2_Te_3_@NPCNFs and Bi_2_Te_3_/BiS_x_@NPCNFs electrodes maintain well‐defined redox peaks with minimal polarization even at high scan rates, suggesting rapid charge‐transfer kinetics.

To further elucidate the charge storage mechanism of the Bi_2_Te_3_@NPCNFs electrode, the b‐values of the anodic and cathodic peaks were determined to be 0.851, 0.888, 0.929, 0.962, 0.885, and 0.766 (Figure 4b), suggesting a pseudocapacitive behavior [46]. Quantitative analysis reveals that the pseudocapacitive contribution of Bi_2_Te_3_@NPCNFs reaches 68.8%, 74.6%, 79.5%, 80.9%, and 83.1% with increasing scan rates, respectively (Figure 4c), significantly surpassing that of Bi_2_Te_3_@NCNFs (62.3%, 69.0%, 74.0%, 76.4%, and 78.5%, Figure S5c). This enhancement is attributed to the engineered Te vacancies and hierarchical porous architecture of Bi_2_Te_3_@NPCNFs, which furnish abundant electrochemically active sites for efficient Na^+^ adsorption. Remarkably, the pseudocapacitive contribution of the Bi_2_Te_3_/BiS_x_@ NPCNFs‐2 electrode was 83.0%, 86.0%, 89.2%, 89.5%, and 91.9%, respectively (Figure 4i), superior to those of similar electrodes (Bi_2_Te_3_@NPCNFs and Bi_2_Te_3_/BiS_x_@NPCNFs‐1 electrodes). This excellent performance originates from the unique crystalline/amorphous Bi_2_Te_3_/BiS_x_ heterostructure, which not only enlarges the active site for Na^+^ storage but also accelerates the ion diffusion kinetics. In addition, the heterointerface synergistically mitigates volume expansion during cycling, ensuring structural integrity and improving long‐term cycling capability.

**FIGURE 5:**
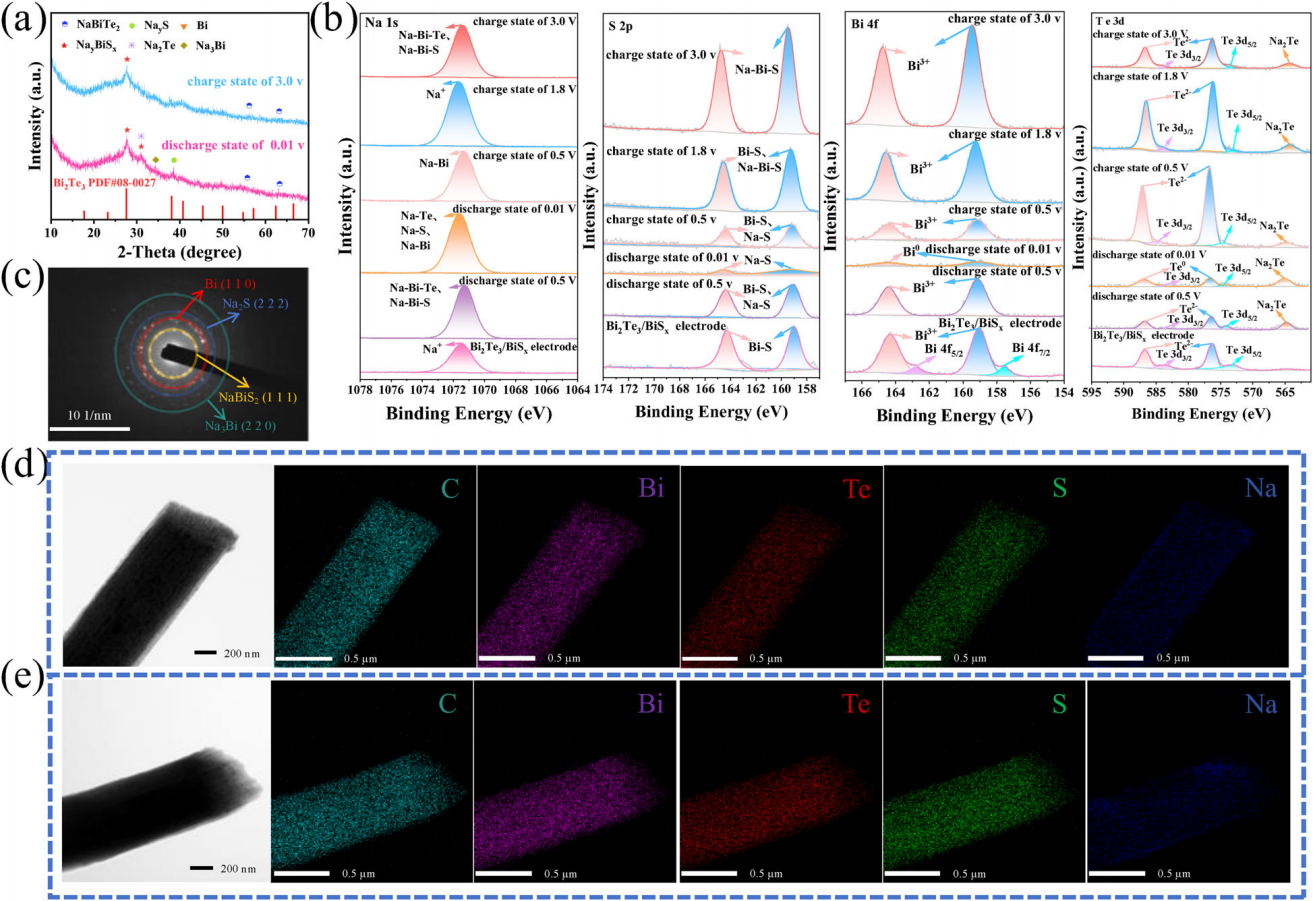
Ex situ (a) XRD patterns, (b) Na 1s, S 2p, Bi 4f, and Te 3d XPS spectra, and (c) SAED of Bi_2_Te_3_/BiS_x_@NPCNFs‐1 at discharge to 0.01 V. Elemental mapping images of Bi_2_Te_3_/BiS_x_@NPCNFs‐1 at (d) discharge to 0.01 V and (e) Charge to 3.0 V.

To reveal the Na^+^ storage mechanism in the crystalline/amorphous Bi_2_Te_3_/BiS_x_@NPCNFs heterostructure, ex situ XRD characterization was performed to track the dynamic phase evolution during sodiation/desodiation (Figure 5a). Upon discharging to 0.01 V, the emergence of diffraction peaks at 27.8° and 31.1° corresponds to the conversion of BiS_x_ into the intermediate phase Na_y_BiS_x_ [47], while concurrent formation of Na_x_Bi_2_Te_3_ signatures indicates the phase transition of Bi_2_Te_3_ to metallic Bi and NaBiTe_2_. Notably, the intermediate Na_y_BiS_x_ undergoes progressive conversion upon further discharge [48]. During the subsequent charge process to 3 V, the reappearance of metallic Bi diffraction peaks accompanied by the disappearance of Na_3_Bi signals confirms the dealloying reaction of Na_3_Bi [19]. The gradual attenuation of both Bi and Na_y_S characteristic peaks, particularly the pronounced decrease in Na_y_S intensity, evidences the reversible conversion between metallic Bi and Na_y_S [49]. The re‐emergence of Na_x_Bi_2_Te_3_ peaks further demonstrates the reversible reaction between Bi and Na_2_Te. Remarkably, after a complete cycle, the Bi_2_Te_3_ phase is fully recovered, as confirmed by the reappearance of its characteristic peaks, albeit with reduced intensity due to partial amorphization [50]. This reversible phase evolution highlights the structural stability of the Bi_2_Te_3_/BiS_x_@NPCNFs heterostructure during repeated Na^+^ insertion/extraction.

Figure 5b presents a comprehensive XPS investigation of the phase transition mechanisms in Bi_2_Te_3_/BiS_x_@NPCNFs‐1 electrodes at various electrochemical states. For the Na 1s spectra, during discharge to 0.5 V, Na^+^ ions progressively incorporate into the lattice, forming both Na‐Bi‐Te and Na‐Bi‐S coordination environments. Upon deeper discharge to 0.01 V, these intermediate phases undergo complete conversion to Na‐Te, Na‐S, and Na‐Bi configurations through a series of reversible electrochemical reactions. The charging process initiates with the oxidation of Na‐Bi alloys back to Na^+^, culminating in the full restoration of the original Na‐Bi‐Te and Na‐Bi‐S bonding configurations at 3.0 V, thereby completing the electrochemical cycle with remarkable reversibility‐a finding that exhibits excellent consistency with the XRD results presented in Figure 5a.

Besides, the evolution of S 2p spectra provides compelling evidence for the bond reorganization dynamics. In the pristine state, sulfur exists predominantly as Bi‐S bonds. The discharge process induces a gradual weakening of Bi‐S interactions concurrent with the emergence of Na‐S species, manifested by a systematic binding energy decrease to 159.2 eV at 0.01 V. Subsequent charging regenerates the Bi‐S bonds while maintaining residual Na‐S configurations and generating new Na‐Bi‐S ternary coordination environments, resulting in an overall binding energy upshift to 159.4 eV due to these synergistic electronic interactions. Bi 4f spectra precisely track the redox chemistry at atomic resolution. The initial Bi^3+^ states (157.5 eV for Bi 4f_7/2_) undergo complete reduction to metallic Bi^0^ (159.1 eV and 164.4 eV) at 0.01 V, accompanied by the transformation of crystalline Bi_2_Te_3_ into amorphous BiS_x_ phases. The charging sequence reveals transient Bi^0^ intermediates before final re‐oxidation to Bi^3+^, demonstrating the electrochemical reversibility of the system. For complementary Te 3d spectra, at low potentials (0.01 V), Te^2^^−^ reduces to both Te^0^ (576.2 eV and 586.5 eV) and Na_2_Te (565–568 eV), with the characteristic 565 eV peak unambiguously assigned to Na_2_Te formation [51]. During the charging phase, Te^2−^ regeneration occurs through Na‐Bi‐Te formation, as evidenced by the corresponding binding energy shifts. This multiscale spectroscopic analysis reveals the complete reaction mechanism of electrochemical phase transitions, confirming that the unique crystalline/amorphous heterostructure promotes excellent reversibility through synergistic bond transitions and controlled phase evolution.

Figure 5c presents selected‐area electron diffraction (SAED) patterns acquired at the fully discharged state (0.01 V), revealing well‐defined diffraction rings corresponding to the reaction products NaBiS_2_ (d‐spacing = 3.33 Å, (111)), metallic Bi (2.27 Å, (110)), Na_2_S (1.87 Å, (222)), and Na_3_Bi (1.36 Å, (220)). These crystalline phases exhibit excellent agreement with the XRD and XPS results, confirming the multi‐step conversion‐alloying mechanism. Remarkably, the corresponding STEM images and EDS elemental mapping (Figure 5d,e) demonstrate exceptional structural integrity of the fibrous architecture after cycling, highlighting the mechanical robustness of the NPCNF matrix in accommodating volume changes during phase transformations. The electrochemical reaction pathway can be delineated as follows:

Discharge process:

Stage I:

$$2Bi_{2}Te_{3}+3Na^{+}+3e^{-}\to 3\mathrm{NaBiT}e_{2}+\mathrm{Bi}$$

$$\mathrm{Bi}S_{x}+\mathrm{yN}a^{+}+ye^{-}\to Na_{y}\mathrm{Bi}S_{x}+\mathrm{Bi}$$

Stage II and VII:

$$\mathrm{NaBiT}e_{2}+3Na^{+}+3e^{-}\to \mathrm{Bi}+2Na_{2}\mathrm{Te}$$

$$Na_{y}\mathrm{Bi}S_{x}+Na^{+}+e^{-}\to Na_{y}S+\mathrm{Bi}$$

Stage III:

$$2\mathrm{Bi}+Na^{+}+e^{-}\to \mathrm{NaB}i_{2}$$

$$\mathrm{NaB}i_{2}+5Na^{+}+5e^{-}\to 2Na_{3}\mathrm{Bi}$$

Charging process:

Stage IV:

$$2Na_{3}\mathrm{Bi}\to \mathrm{NaB}i_{2}+5Na^{+}+5e^{-}$$

Stage V:

$$\mathrm{NaB}i_{2}\to 2\mathrm{Bi}+Na^{+}+e^{-}$$

Stage VI:

$$\mathrm{Bi}+2Na_{2}\mathrm{Te}\to \mathrm{NaBiT}e_{2}+3Na^{+}+3e^{-}$$

$$Na_{y}S+\mathrm{Bi}\to Na_{y}\mathrm{Bi}S_{x}+Na^{+}+e^{-}$$

**FIGURE 6:**
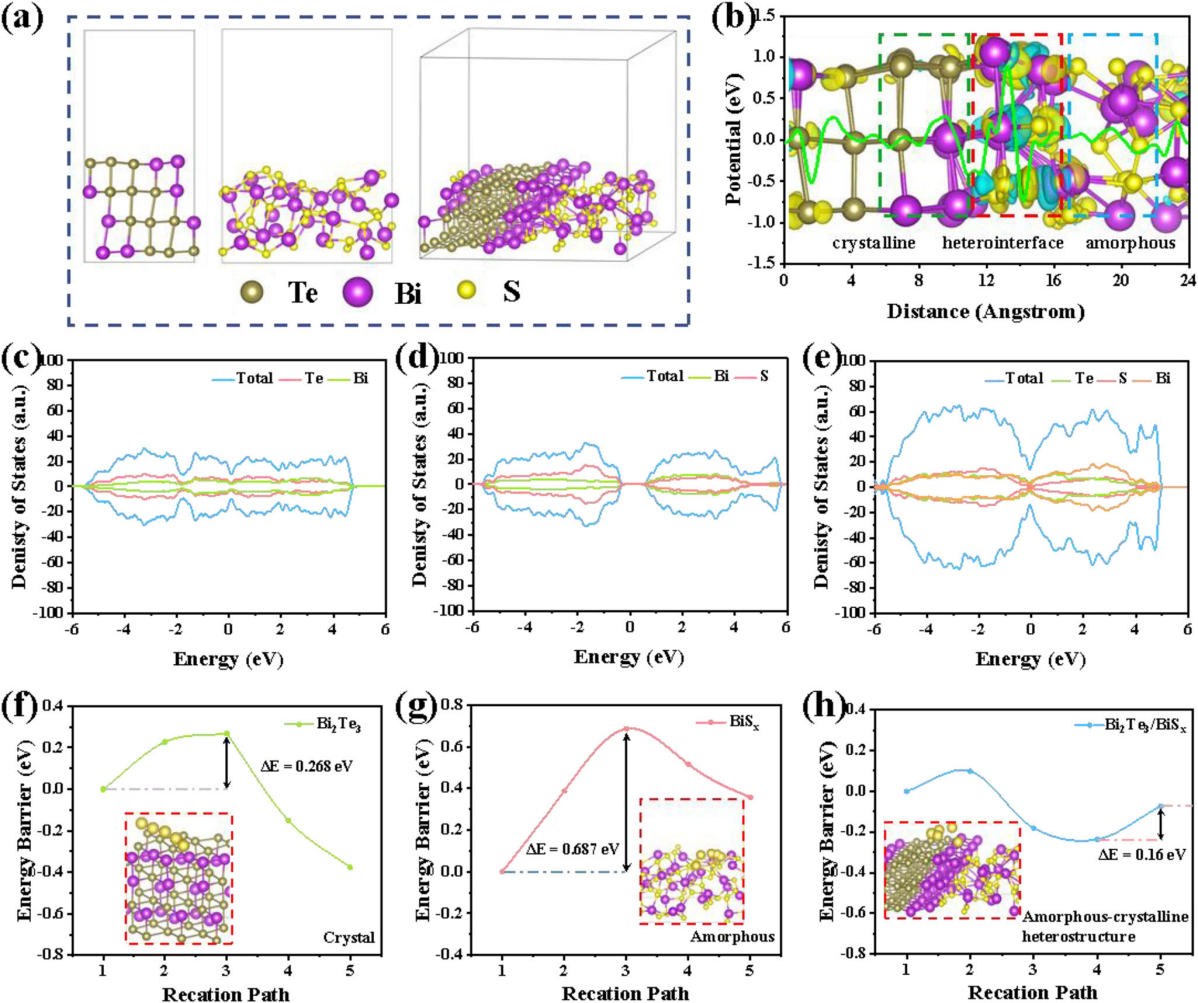
(a) The Bi_2_Te_3_, BiS_x_, and Bi_2_Te_3_/BiS_x_ heterostructure models. (b) Charge density difference of the amorphous‐crystalline interface. (c–e) Density of states (DOS) of Bi_2_Te_3_, BiS_x_, and Bi_2_Te_3_/BiS_x_ heterostructure. (f) Crystalline structure, (g) amorphous structure, and (h) amorphous‐crystalline structure. Insets show the diffusion path.

To theoretically reveal the outstanding dynamic properties of heterogeneous structures, the density functional theory (DFT) calculations were employed. Figure 6a illustrates the structural models of crystalline Bi_2_Te_3_, amorphous BiS_x_ [52, 53], and Bi_2_Te_3_/BiS_x_ heterostructure. Through precise electronic structure analysis, the charge transfer dynamics were detailed, as shown in Figure 6b. Additionally, the charge redistribution at the crystal/amorphous interface exhibits a distinct electron transfer phenomenon, spanning the crystal/amorphous heterointerface. From the planar average charge density distribution along a certain direction, localized charge accumulation and depletion phenomena are observed, as indicated by the dashed lines. Due to this localized charge transfer, an internal electric field and a space charge region are induced, thereby accelerating the mobility of charge carriers and the electrochemical activity of the electrodes [54, 55]. Electronic conductivity is a critical factor in determining the electrochemical performance of anode materials. Therefore, a comprehensive investigation of the electronic structure was conducted, including the calculation of the density of states (DOS) under different structural configurations (Figure 6c–e). The total density of states (TDOS) and integrated density of states (DOS) values for Bi_2_Te_3_/BiS_x_ indicate an increased number of electronic states near the Fermi level, leading to elevated carrier concentration. This explains why Bi_2_Te_3_/BiS_x_ exhibits superior conductivity, attributed to its crystalline/amorphous heterostructure. Furthermore, the ion diffusion energy barriers along multiple pathways were calculated for different structural models (Figure 6f–h). Notably, Bi_2_Te_3_ has a bandgap of 0.268 eV, while BiS_x_ has a bandgap of 0.687 eV. Upon constructing the Bi_2_Te_3_/BiS_x_ heterostructure, the bandgap is reduced to 0.16 eV, resulting in significantly enhanced conductivity [56]. These findings highlight the importance of the Bi_2_Te_3_/BiS_x_ heterostructure formation in enhancing overall electronic conductivity.

Building upon the exceptional electrochemical properties of the rationally designed Bi_2_Te_3_/BiS_x_ heterostructure@NPCNFs anode material, we demonstrate its successful integration into a full‐cell configuration with Na_3_V_2_(PO_4_)_3_ (NVP) cathodes, as illustrated in Figure S7a. To achieve optimal charge balance in the Bi_2_Te_3_/BiS_x_@NPCNFs//NVP full cell configuration, the negative‐to‐positive electrode capacity ratio (N/P ratio) was precisely calibrated to 1.1. The loading of active material onto the working electrode was precisely controlled. In the working electrode for electrochemical testing, the loading of active material was precisely maintained between 1.66 and 1.87 mg. The operational voltage window of the full cell was established within the range of 0.4 to 3.6 V to ensure stable electrochemical performance and prevent overcharge or overdischarge degradation. Prior to the assembly of the full cell, the electrode sheets were subjected to a pre‐sodium treatment procedure. This pre‐treatment was carried out either by mechanically pressing the electrodes or by subjecting them to a pre‐activation electrochemical process. Following this step, the treated electrode sheets were carefully extracted and subsequently utilized in the assembly of the complete sodium‐ion full cell. The assembled full cell delivers outstanding electrochemical performance, maintaining 85% capacity retention after 100 cycles at 0.1C (Figure S7b), achieving remarkable energy and power densities of 322 Wh kg^−1^ and 225 W kg^−1^, respectively. The system sustains exceptional coulombic efficiency exceeding 98.4% throughout extended cycling (Figure S7c), with a stable operating voltage plateau between 2.0 and 2.5 V (Figure S7d,e) enabled by optimal potential matching between the low‐voltage Bi‐based anode and high‐voltage NVP cathode.

Impressively, the Bi_2_Te_3_/BiS_x_@NPCNFs//NVP configuration demonstrates superior electrochemical storage properties compared to state‐of‐the‐art sodium‐ion full cells (Figure S7f) [57, 58, 59, 60]. The superior performance originates from several synergistic design features: (i) the 3D porous N‐doped carbon nanofiber matrix provides both mechanical support and efficient charge transport pathways, (ii) the precisely engineered Bi_2_Te_3_/BiS_x_ heterointerfaces enable rapid Na^+^ diffusion and electron transfer, (iii) the unique crystalline‐amorphous dual‐phase architecture ensures structural integrity during repeated sodiation/desodiation. This successful full‐cell validation confirms the practical viability of our heterostructure design concept. Furthermore, it establishes a new materials paradigm for advanced sodium‐ion batteries, thereby offering exciting opportunities for developing next‐generation energy storage systems.

## 3 Conclusions

In summary, we demonstrate a rationally designed crystalline/amorphous Bi_2_Te_3_/BiS_x_ heterostructure encapsulated in N‐doped carbon nanofibers (Bi_2_Te_3_/BiS_x_ @NPCNFs) as an advanced anode material for sodium‐ion batteries, synthesized via an innovative in situ simultaneous tellurization/sulfidation strategy. The precisely engineered architecture combines crystalline Bi_2_Te_3_ domains for efficient electron transport, amorphous BiS_x_ matrices for strain accommodation, and a conductive N‐doped carbon network for structural stability, creating abundant electrochemically active interfaces. The synergistic effects of this unique heterostructure design, combined with Te vacancy engineering and hierarchical porosity, enable exceptional sodium storage performance through optimized conversion‐alloying mechanisms, delivering a superior rate capability (341.6 mAh g^−1^ after 100 cycles), and excellent long‐term cyclic stability (288.7 mAh g^−1^ at 1.0 A g^−1^ after 1000 cycles) and remarkable cycling stability (94.2% capacity retention after 100 cycles). Comprehensive electrochemical characterization and ex situ spectroscopic and microscopic studies reveal the reversible phase evolution and reaction pathways during sodiation/desodiation processes, revealing the critical role of defect engineering and interfacial effects in enhancing Na^+^ storage kinetics. The practical viability is further confirmed in a full‐cell configuration with Na_3_V_2_(PO_4_)_3_ cathodes, achieving an impressive energy density of 322 Wh kg^−1^ and stable cycling stability. This study establishes a general materials engineering strategy for developing advanced energy storage systems, offering valuable insights for next‐generation battery technologies requiring both high energy density and ultra‐long cycle life.

## Conflicts of Interest

The authors declare no conflicts of interest.

## Supporting information




**Supporting File**: smll73235‐sup‐0001‐SuppMat.docx.

## Data Availability

The data that support the findings of this study are available from the corresponding author upon reasonable request.
