# Supplementary material for: Chalcogen‐Directed Dual‐Heterointerfaced Bi2Te3/BiSx in N‐Doped Carbon Nanofibers for Sodium Storage
Source: Small. 2026 Mar 26;22(28):e73235. doi: 10.1002/smll.73235 (PMC13181519; doi:10.1002/smll.73235)
Supplement: Supplementary file 1 — Supporting File: smll73235‐sup‐0001‐SuppMat.docx. [file SMLL-22-e73235-s001.docx]

Supporting Information

**Chalcogen-Directed Dual-Heterointerfaced Bi₂Te₃/BiSₓ in N-Doped Carbon Nanofibers for Sodium Storage**

Jinkai Wang^a*,†^, Min Wang^a†^, Hangcheng Yang^b†^, Zhengdong Wang^a^, Yaoyao Wang^a^, Gengrui Liu^a^, Yiming Yong^a^, Boshi Liu^a^, Xinrui Zhou^a^, Hongdu He^a^, Hongkang Wang^b*^, Zongyou Yin^c*^

^a^ School of Mechanical and Electrical Engineering, Xi’an University of Architecture and Technology, Xi’an 710055, China

^b^ State Key Lab of Electrical Insulation and Power Equipment, Center of Nanomaterials for Renewable Energy (CNRE), School of Electrical Engineering, Xi'an Jiaotong University, Xi’an 710049, China

^c^ Research School of Chemistry, Australian National University, Canberra, ACT, 2601 Australia

**Experimental Section**

**Preparation of Bi_2_Te_3_@NPCNFs Composites**

The Bi_2_Te_3_@NPCNFs composites were fabricated through an optimized electrospinning and thermal treatment approach. Initially, a homogeneous precursor solution was prepared by dissolving polyacrylonitrile (PAN, average MW 149000~151000, 1.2 g), polyvinylpyrrolidone (PVP, average MW 1300000, K88-96, 0.6 g), and dicyandiamide (DCD, 98%, 0.3 g) in N,N-dimethylformamide (DMF, 99.8%, 12 mL) under continuous magnetic stirring at 50°C for 1 h. Subsequently, bismuth nitrate pentahydrate (Bi(NO_3_)_3_·5H_2_O, 98.0%, 1.2 g) was introduced into the solution and vigorously stirred for 3 h, yielding a homogeneous beige solution.

For electrospinning, the solution was loaded into a 10 mL syringe equipped with a stainless-steel needle. The electrospinning process was conducted under optimized conditions with flow rate of 16.7 μL/min, applied voltage of 16 kV, and collector rotation speed of 300 rpm, yielding flexible white PAN-PVP-Bi nanofibers. The as-prepared precursor nanofibers were dried at 70°C for 24 h in a vacuum oven before undergoing a two-step thermal treatment: pre-oxidation in air at 280°C (heating rate: 10°C/min) for 1 h, followed by carbonization at 600°C for 2 h under argon atmosphere (heating rate: 10°C/min) in the presence of tellurium powder with a mass ratio of PAN-PVP-Bi nanofibers and Te powder (99.9%) to be 1:2, ultimately producing black Bi_2_Te_3_@NPCNFs composites.

For comparative studies, control samples were prepared through similar procedures. The Bi_2_Te_3_@NCNFs material was synthesized under identical experimental conditions but without PVP addition in the precursor solution.

**Preparation of Bi_2_Te_3_/BiS_x_@NPCNFs Composites**

The obtained Bi_2_Te_3_@NPCNFs were further sulfurized in a tube furnace at 600°C for 2 hours with different S powder (99.9%) to Bi_2_Te_3_@NPCNFs mass ratios to ultimately yield the crystalline/amorphous Bi_2_Te_3_/BiS_x_ heterostructures@carbon matrix (Bi_2_Te_3_/BiS_x_@ NPCNFs-x) composites, where x represents the mass ratio of S to Bi_2_Te_3_@NPCNFs (Bi_2_Te_3_/BiS_x_@NPCNFs-1: the mass ratio of S to Bi_2_Te_3_@NPCNFs is 1:2; Bi_2_Te_3_/BiS_x_@NPCNFs-2: the mass ratio of S to Bi_2_Te_3_@NPCNFs is 2:1).

**Structural Characterization**

The morphology and microstructure of the samples were investigated using scanning electron microscopy (SEM, Hitachi Regulus8100) and transmission electron microscopy (TEM, JEOL-JEM 2100F), with high-resolution TEM (HRTEM) employed for detailed lattice analysis. Crystallographic phases were characterized by X-ray diffraction (XRD, Bruker D8 Advance diffractometer), while chemical states were analyzed using X-ray photoelectron spectroscopy (XPS, Thermo Scientific ESCALAB Xi+). Raman spectroscopy (LabRam HR Evolution) was used to evaluate the graphitic structure, and thermal stability was assessed via thermogravimetric analysis (TGA, METTLER TOLEDO TGA/DSC 1) under controlled atmosphere.

**Electrochemical Measurements**

The working electrodes were prepared by thoroughly mixing the active materials (Bi_2_Te_3_@NCNFs, Bi_2_Te_3_@NPCNFs, Bi_2_Te_3_/BiS_x_@NPCNFs-1, and Bi_2_Te_3_/BiS_x_@ NPCNFs-2), conductive carbon black, and polyacrylic acid (PAA) binder in a mass ratio of 7:2:1 through mechanical grinding for 1 h. The resulting homogeneous slurry was uniformly coated onto copper foil and subsequently dried at 60°C for 12 h in a vacuum oven. CR2032 coin cells were assembled in an argon-filled glove box (<0.1 ppm H_2_O/O_2_), employing sodium metal as the counter electrode, the prepared composite electrodes as the working electrodes, and 1 M NaClO_4_ in PC additive as electrolyte.

**Computational Methods**

All density functional theory (DFT) calculations were performed using the Vienna Ab initio Simulation Package (VASP) [[1](#_ENREF_1)]. The electron-ion interactions were treated with the projector-augmented wave (PAW) method [[2](#_ENREF_2)], while the exchange-correlation functional was approximated by the generalized gradient approximation (GGA) in the Perdew-Burke-Ernzerhof (PBE) formulation. To account for van der Waals interactions, which are poorly described by standard GGA functionals, we incorporated Grimme's DFT-D3 dispersion correction [[3](#_ENREF_3),[4](#_ENREF_4)]. The self-consistent solution of the Kohn-Sham equations was converged to an energy tolerance of 10^-5^ eV. For structural optimization of the Bi_2_S_3_, Bi_2_Te_3_, and Bi_2_S_3_-Bi_2_Te_3_ surface models, we employed Gamma-centered k-mesh with grid densities of 3×3×1 for Bi_2_S_3_ and Bi_2_Te_3_, and 1×1×1 for the heterostructure. During geometry relaxation, the bottom three atomic layers were fixed to simulate bulk-like behavior, while remaining atoms were allowed to relax until atomic forces fell below 0.03 eV/Å. Subsequent electronic structure analysis utilized denser k-meshes to ensure accurate density of states (DOS) calculations: 5×4×1 for Bi_2_S_3_, 5×5×1 for Bi_2_Te_3_, and 2×2×1 for the Bi_2_S_3_-Bi_2_Te_3_ heterostructure. Ion diffusion energetics were investigated using the climbing image nudged elastic band (CI-NEB) method [[5](#_ENREF_5),[6](#_ENREF_6)], which provides reliable minimum-energy pathways and activation barriers for atomic migration processes.

**Fig. S1.** (a,b) SEM images of the Bi_2_Te_3_@NCNFs.

**Fig. S2.** (a) XRD patterns, (b) Raman spectra , and (c) TGA of Bi_2_Te_3_@NCNFs. XPS spectra of Bi_2_Te_3_@NCNFs (d) Survey spectrum, (e) C 1s , (f) N 1s, (g) Te 3d, and (h) Bi 4f core-level spectra.

**Fig. S3.** (a-c) CV curve at 0.2 mV s^-1^, (d-f) GCD profiles at different current densities, and (g-i) GCD profiles at 0.1 A g^-1^ of Bi_2_Te_3_@NCNFs, Bi_2_Te_3_@NPCNFs and Bi_2_Te_3_/BiS_x_@NPCNFs-1 electrodes.

**Fig. S4.** (a) Rate capability at various rates, (b) Cycling performances at 0.1 A g^-1^, (c) 0.5 A g^-1^ and (d) 1.0 A g^-1^ of Bi_2_Te_3_@NCNFs electrode. (e) EIS spectra after 10 cycles at 0.1 A g^-1^, (f) Linear diagram of impedance and frequency components in (e), and (g) GITT curve, and (h) Diffusion coefficients calculated from GITT curve of Bi_2_Te_3_@NCNFs electrode.

**Fig. S5.** (a) CV curves at different scan rates, (b) calculated b values from CV curves, and (c) Capacitive contribution at different scan rates of Bi_2_Te_3_@NCNFs electrode.

**Fig. S6.** (a) XRD patterns, (b) Rate capability at various rates, (c) Cycling performances at 0.5 A g^-1^ of BiS_x_.

**Fig. S7.** Electrochemical behaviors of Bi_2_Te_3_/BiS_x_@NPCNFs//NVP sodium ion full cells batteries. (a) The schematic Bi_2_Te_3_/BiS_x_@NPCNFs//NVP full cell. (b) Rate performance. (c) Cycling performances at 0.1 A g^-1^ and inset optical photograph of the powered thermograph. (d) GCD curves at different current densities. (e) GCD curves at 0.1 A g^-1^. (f) Comparison of the reported materials in power density and energy density of full cells.

**Table S1.** Summary of the electrochemical performance of Bi-based anode materials in SIBs.

| **Materials** | **Methods** | **Capacity** | **Cyclic stability** | **Refs** |
| --- | --- | --- | --- | --- |
| Bi_0.4_Sb_0.6_ | One step co-replacement reaction | 290 mAh g^-1^  (0.5 A g^-1^) | 1 A g^-1^, 700 cycles, 256.1 mAh g^-1^ | [[7](#_ENREF_7)] |
| Bi_2_Te_3_@PPy | Solvothermal Method | 357 mAh g^-1^  (0.1 A g^-1^) | 2 A g^-1^, 200 cycles, 209 mAh g^-1^ | [[8](#_ENREF_8)] |
| Bi_2_Se_3_/Bi_2_O_3_ heterostructure | Hydrothermal method and annealing process | 352 mAh g^-1^  (0.1 A g^-1^) | 0.1 A g^-1^, 100 cycles, 310 mAh g^-1^ | [[9](#_ENREF_9)] |
| Graphene−loaded  Bi_2_Se_3_ | Selenization | 346 mAh g^-1^  (0.1 A g^-1^) | 0.1 A g^-1^, 90 cycles,  260 mAh g^-1^ | [[10](#_ENREF_10)] |
| np-Bi_6_Sb_2_ | Alloying | 258 mAh g^-1^  (0.2 A g^-1^) | 1 A g^-1^, 10000 cycles, 150 mAh g^-1^ | [[11](#_ENREF_11)] |
| Bi_2_Se_3_-C/G | Lyophilization, carbonization and Selenization | 260 mAh g^-1^  (0.1 A g^-1^) | 2 A g^-1^, 1000 cycles, 240 mAh g^-1^ | [[12](#_ENREF_12)] |
| Bi_2_Te_3_/BiS_x_  @NPCNFs | Electrospinning,  Tellurization  and Sulfurization | 367.7 mAh g^-1^  (0.1 A g^-1^) | 1 A g^-1^, 1000 cycles, 288.7 mAh g^-1^ | This work |

**References**

[1] J. Hafner, Ab‐initiosimulations of materials using VASP: Density‐functional theory and beyond, J. Comput. Chem. 29(13) (2008) 2044-2078, <https://doi.org/10.1002/jcc.21057>.

[2] P.E. Blöchl, Projector augmented-wave method, Phys. Rev. B 50(24) (1994) 17953-17979, <https://doi.org/10.1103/PhysRevB.50.17953>.

[3] S. Grimme, J. Antony, S. Ehrlich, H. Krieg, A consistent and accurateab initioparametrization of density functional dispersion correction (DFT-D) for the 94 elements H-Pu, J. Chem. Phys. 132(15) (2010) 154104, <https://doi.org/10.1063/1.3382344>.

[4] S. Grimme, S. Ehrlich, L. Goerigk, Effect of the damping function in dispersion corrected density functional theory, J. Comput. Chem. 32(7) (2011) 1456-1465, <https://doi.org/10.1002/jcc.21759>.

[5] G. Henkelman, H. Jónsson, Improved tangent estimate in the nudged elastic band method for finding minimum energy paths and saddle points, J. Chem. Phys. 113(22) (2000) 9978-9985, <https://doi.org/10.1063/1.1323224>.

[6] G. Henkelman, B.P. Uberuaga, H. Jónsson, A climbing image nudged elastic band method for finding saddle points and minimum energy paths, J. Chem. Phys. 113(22) (2000) 9901-9904, <https://doi.org/10.1063/1.1329672>.

[7] Y. Liu, X. Liu, X. Wang, S. Ullah, Y. Peng, G. Pan, W. Gao, B. Song, X. Zhang, A. Jia, Synergistic bimetallic effects of BiSb anodes enable long‐stable sodium storage, Adv. Funct. Mater. 35(6) (2025) 2415092, <https://doi.org/10.1002/adfm.202415092>.

[8] X. Liu, Y. Si, K. Li, Y. Xu, Z. Zhao, C. Li, Y. Fu, D. Li, Exploring sodium storage mechanism of topological insulator Bi_2_Te_3_ nanosheets encapsulated in conductive polymer, Energy Storage Mater. 41 (2021) 255-263, <https://doi.org/10.1016/j.ensm.2021.06.004>.

[9] M. Han, Z. Zhou, Y. Li, Q. Chen, M. Chen, Constructing Bi_2_Se_3_/Bi_2_O_3_ heterostructure as promising anode for efficient sodium-ion storage, J. Alloys Compd. 892 (2022) 162143, <https://doi.org/10.1016/j.jallcom.2021.162143>.

[10] D. Li, J. Zhou, X. Chen, H. Song, Graphene-loaded Bi_2_Se_3_: a conversion–alloying-type anode material for ultrafast gravimetric and volumetric Na storage, ACS Appl. Mater. Interfaces 10(36) (2018) 30379-30387, <https://doi.org/10.1021/acsami.8b09538>.

[11] H. Gao, J. Niu, C. Zhang, Z. Peng, Z. Zhang, A dealloying synthetic strategy for nanoporous bismuth–antimony anodes for sodium ion batteries, ACS Nano 12(4) (2018) 3568-3577, <https://doi.org/10.1021/acsnano.8b00643>.

[12] D. Li, J. Hu, C. Wang, L. Guo, J. Zhou, Metal-organic framework-induced edge-riched growth of layered Bi_2_Se_3_ towards ultrafast Na-ion storage, J. Power Sources 555 (2023) 232387, <https://doi.org/10.1016/j.jpowsour.2022.232387>.
